# Large Water-Clear-Cell Parathyroid Adenoma: A Report of a Rare Case

**DOI:** 10.7759/cureus.44158

**Published:** 2023-08-26

**Authors:** Olivia Durant, Zan Ahmed, Muhammad Tahir, Kurt Knowles, Joe S Liles

**Affiliations:** 1 Pathology and Laboratory Medicine, University of South Alabama Health Hospital, Mobile, USA; 2 Surgery, University of South Alabama Health Hospital, Mobile, USA

**Keywords:** parathyroid neoplasms, parathyroid pathology, parathyroid disorder, water clear cell parathyroid adenoma, parathyroid gland adenoma

## Abstract

Water-clear-cell parathyroid adenomas are extremely rare tumors characterized by cells that contain clear, foamy cytoplasm. Here we report a case of a large water-clear-cell parathyroid adenoma in a 70-year-old male. The patient was presented to an outside hospital with severe abdominal pain and supporting CT imaging confirming a small bowel obstruction. Initial laboratory studies revealed hypercalcemia and elevated parathyroid hormone levels. Subsequent ultrasound imaging revealed a 2.7 × 2.1 cm neck mass suspicious for a parathyroid adenoma. A parathyroidectomy was performed, and microscopic evaluation revealed an expansile proliferation of cells with characteristic water-clear cell features. Although rare, water-clear-cell parathyroid adenomas are clinically indistinguishable from more common subtypes and should be considered in the differential diagnosis of an anterior neck mass.

## Introduction

Parathyroid adenoma is the most common cause of primary hyperparathyroidism, a condition that affects nearly 1% of adults [1]. These benign tumors produce an excess of parathyroid hormone (PTH), which in turn leads to hypercalcemia and its associated signs and symptoms of gastrointestinal upset, bone pain, renal stones, polyuria, polydipsia, fatigue, confusion, depression, and memory loss. Histologically, most parathyroid adenomas are composed of hypercellular proliferation of chief cells, oxyphilic cells, or oncolytic cells [2,3]. An adenoma consisting of water-clear cells (WCCs) is an extremely rare subtype, with fewer than 40 cases reported in English literature [4]. Here we present the case of a 70-year-old male who was diagnosed with a WCC parathyroid adenoma presenting with intestinal obstruction.

## Case presentation

**Figure 1:**
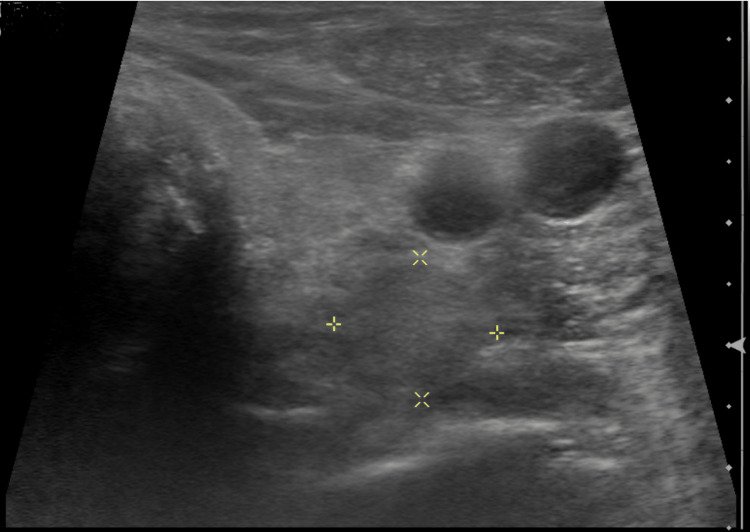
An ultrasound of the neck shows a solid area located lateral and inferior to the left thyroid gland.

**Figure 2:**
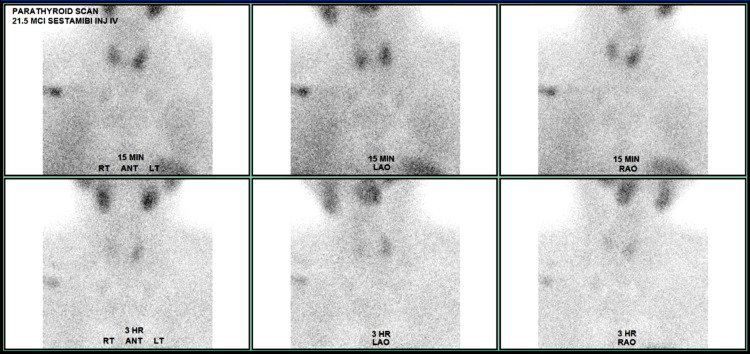
Technetium-99m Sestamibi (MIBI) scan showed a significant increase in uptake after 3 hours.

A 70-year-old male initially presented to an outside hospital with symptoms of sharp abdominal pain, nausea, and vomiting. He was vitally stable upon admission, and supporting CT imaging revealed a small bowel obstruction. Initial lab results revealed hypercalcemia, with a total serum calcium of 12.1 mg/dL. Further workup revealed a PTH level of 119.3 pg/mL, and he was diagnosed with primary hyperparathyroidism. The patient’s hypercalcemia improved after fluid administration, and his bowel obstruction was resolved with conservative management. He was discharged on the third day of hospitalization. Subsequent ultrasound imaging revealed a 2.7 × 2.1 cm lesion lateral and inferior to the left thyroid gland, and a technetium-99m sestamibi (MIBI) scan was significant for uptake at 3 hours in the left neck, suggesting a possible parathyroid adenoma (Figures 1, 2). Upon further questioning, the patient endorsed common signs and symptoms of hypercalcemia, such as bone pain, kidney stones, abdominal pain, insomnia, and forgetfulness. He denied any family history of hyperparathyroidism or other endocrine malignancies. Additional medical history includes osteoporosis, hypertension, Barrett’s esophagus, and prior cholecystectomy. The patient endorsed the current use of tobacco products along with daily alcohol consumption. The patient was referred to USA Health Surgical Oncology for further evaluation. After counseling on management options, informed consent was obtained for a surgical parathyroidectomy.

On the day of operation, morning laboratory results yielded a total serum calcium of 12.7 mg/dL (8.5-10.1 mg/dL) and an intact PTH of 110.4 pg/mL (13.8-85.0 pg/mL). After appropriate time-out and anesthesia, the patient was placed in a supine position with neck extension and neuromonitoring, and the left lateral thyroid was mobilized, providing a view to the left superior parathyroid tissue. The tissue was excised and passed off to pathology for frozen section evaluation. Intraoperatively, frozen section results confirmed the presence of hypercellular parathyroid tissue, and the specimen was placed in formalin for permanent. The recurrent laryngeal nerve was identified and preserved via neuromonitoring during the operation. There were no intraoperative or postoperative complications. At the two-week follow-up appointment, the patient was feeling well and denied any symptoms of hypocalcemia. Laboratory values demonstrated a correction of total serum calcium at 9.7 mg/dL and a lower intact PTH level of 11.4 pg/mL, which can occur after the removal of a large parathyroid adenoma. The patient was advised to return to the clinic in two months for an additional follow-up appointment.

Pathological examination

**Figure 3:**
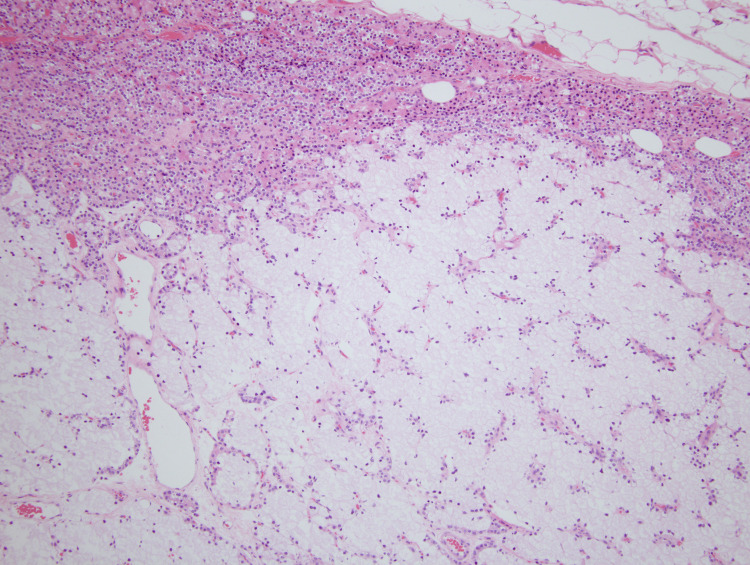
A low-power view outlining the parathyroid adenoma composed of predominantly clear cells; a small portion of residual non-neoplastic parathyroid tissue is seen, consisting predominately of chief cells (10×).

**Figure 4:**
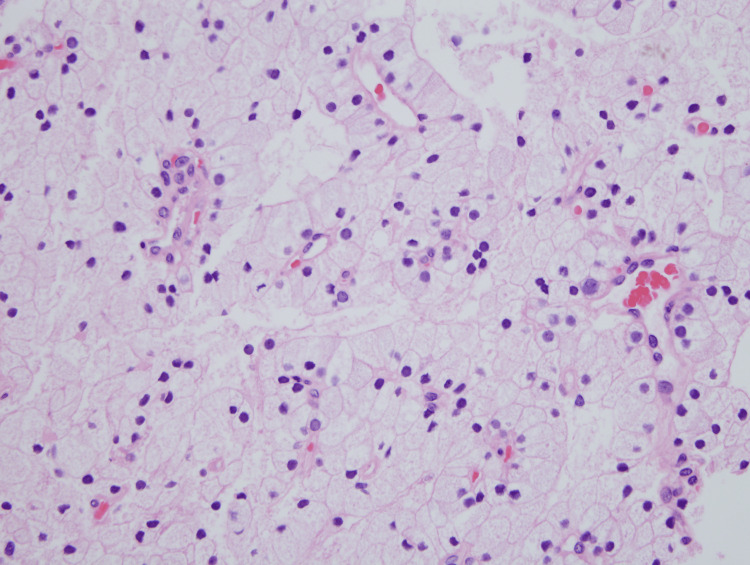
High-power view demonstrating water-clear cells with abundant clear cytoplasm and an overall bland architecture (40×).

**Figure 5:**
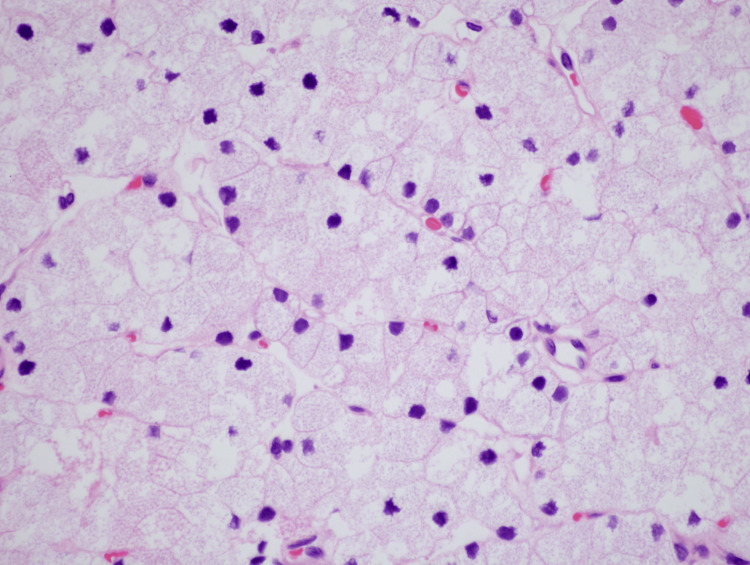
High-power view illustrating characteristic features of large cells with optically clear cytoplasm and sharply outlined cell membranes with no significant pleomorphism or mitotic activity (60×).

A gross examination of the resected specimen revealed a 2.886 g, 3.2 x 1.5 x 0.6 cm, tan-encapsulated tissue fragment covered by adipose tissue. Upon sectioning, the cut surfaces demonstrated a cystic area filled with brown serous fluid. Microscopically, the tissue revealed an expansile proliferation of predominately WCCs with no significant atypia or mitotic activity (Figures 3-5). The cystically dilated area showed degenerative changes with minimal preservation of cell boundaries. Overall, the architecture was bland and lacked significant mitotic activity or atypia. Based on histological features, the diagnosis of parathyroid adenoma with WCCs was rendered.

## Discussion

Primary hyperparathyroidism is one of the most common endocrine disorders, and it is an important cause of hypercalcemia. Notably, adenomas are the most frequently cited etiology, comprising up to 75-80% of cases, followed by primary hyperplasia and carcinoma [5]. Primary hyperparathyroidism is usually a disease of adults (fourth decade of life) with a female predominance (3:1).

Clinically, hyperparathyroidism can be divided into two categories: symptomatic and asymptomatic. "Painful bones, renal stones, abdominal groans, and psychic moans" are terms that have been used to describe the spectrum of symptoms. Biochemically, the most common manifestation of primary hyperparathyroidism is an increase in the level of serum ionized calcium [6]. The serum abnormalities may lead to nephrolithiasis, gastrointestinal disturbances such as constipation, and neuromuscular complaints manifesting as weakness and fatigue.

Most adenomas are solitary masses that are grossly oval to slightly lobulated with a thin connective tissue capsule. On the cut section, they are often grayish brown with possible foci of hemorrhage, calcification, or cystic change, with an increased predilection to involve one of the lower glands. 

Microscopically, adenomas are encapsulated and highly cellular. An adjacent rim of compressed non-neoplastic tissue can be identified in about 60% of cases. Various cell types may comprise the adenoma, but chief cells usually predominate. Mitotic figures are usually absent, and the pattern of growth is diffuse, or to a lesser extent, nesting, follicular, or pseudopapillary [7]. 

In our case, WCCs predominated. The cells are large, optically clear, and have sharply outlined cell membranes. Grossly, water-clear variant adenomas have a predilection to involve the superior glands, are soft, and are typically chocolate brown in color. Cysts and hemorrhages have been observed. Interestingly, a strong association with blood group O has been observed [8].

The WCC adenoma is an extremely rare subtype of parathyroid adenoma. A recent literature review claims that only 37 cases have been reported in the English literature between 1985 and 1981, with most occurring in middle-aged women [6]. WCCs are not considered part of normal parathyroid architecture. They are hypothesized to originate from aged Golgi vesicles or granular endoplasmic reticulum cisterns and have been associated with both parathyroid hyperfunction and increasing age [9]. It cannot be distinguished clinically from a classical adenoma, and histopathology is often required to support the diagnosis. Like classic adenomas, they are usually contained in a fibrous capsule, which distinguishes them from WCC hyperplasia, a histologically similar entity that affects less than 1% of the population. However, they are larger in size and weight [10].

Histologically, WCC adenomas are composed of large polygonal cells with abundant clear cytoplasm that contain vacuoles with or without granular material. This composition may cause them to appear isoechoic on ultrasound imaging and have reduced scintigraphy sensitivity [11]. Immunohistochemical analysis should be performed to confirm WCC adenoma and rule out the metastatic disease with a similar microscopic appearance, such as renal clear cell carcinoma. WCC adenomas will express diffuse positivity for PTH and nuclear positivity for GATA3 [12]. WCC adenomas have low endocrine activity, and despite secreting large amounts of PTH, calcium levels will remain within normal limits unless considerable size is reached, as in our case presentation. Malignant transformation is exceedingly rare [13].

## Conclusions

In summary, we report a rare case of large WCC parathyroid adenoma successfully treated with surgical resection without residual signs of hypercalcemia. Our patient initially presented with abdominal pain secondary to small bowel obstruction in the setting of PTH-dependent hypercalcemia. Further work-up revealed a parathyroid adenoma with histopathologic confirmation of a WCC variant. Although less aggressive, WCC adenomas can present with a myriad of symptoms. This is a rare entity and, to our knowledge, the first reported case in the state of Alabama.
